# Barriers to Treatment for Female Problem Gamblers: A UK Perspective

**DOI:** 10.1007/s10899-016-9663-1

**Published:** 2016-12-22

**Authors:** Anna Kaufman, Jessica D. Jones Nielsen, Henrietta Bowden-Jones

**Affiliations:** 1 Department of Psychology, City University London, Northampton Square, London, EC1V 0HB UK; 2 Department of Medicine, Faculty of Medicine, Imperial College, London, SW7 2AZ UK

**Keywords:** Problem gambling, Female, Treatment, Barriers, Interpretative phenomenological analysis, Cognitive-behavioural therapy

## Abstract

There is a paucity of research in the UK which examines problem gambling and that which does exist is mainly quantitative, focuses on male samples and fails to look at treatment seeking populations or obstacles preventing problem gamblers from seeking treatment. This paper presents findings from part of a larger qualitative study that explored the experience of treatment for female problem gamblers. Data were collected using semi-structured interviews with eight women who had received individual cognitive-behavioural therapy in the National Health Service for their gambling problem. An interpretative phenomenological analysis approach was applied in the research process, identifying three main themes, of which the subtheme ‘Barriers to Treatment’ is examined here. Internal and external barriers to treatment organically emerged in all female participants’ accounts and appear to have an impact on service utilisation. Input directly from gamblers can be combined with findings from other studies to devise better ways of reaching female problem gamblers. A better understanding of barriers to treatment can also provide valuable direction for future research and suggest applications in clinical service provision and treatment planning.

## Introduction

In recent years the gambling landscape in Britain has changed significantly. The introduction of the Gambling Act 2005, which came into effect in 2007, has allowed the responsible advertising of gambling and the addition of licensed online gambling, making it possible for problem gamblers to access gaming facilities 24 h a day. While gambling has traditionally been perceived as a male recreational activity (Potenza et al. 2001) and a male addiction, recent gambling prevalence studies indicate the gap is narrowing as a greater number of women are now gambling (Wardle and Seabury 2013; Wardle et al. 2011). Yet little research has focused on female problem gamblers and, in particular, aspects of the treatment-seeking experience for women has been neglected.

According to the latest British Gambling Prevalence Survey, commissioned by the Gambling Commission, which regulates gambling activities in the UK, there are between 360,000 and 450,000 adult problem gamblers in Great Britain. The prevalence of male problem gambling increased from 1% in 2007 to 1.5% in 2010, while female problem gambling increased from 0.2% in 2007 to 0.3% in 2010, which translates to 75,000 women in Great Britain (Wardle et al. 2011). Interestingly, the increase in overall prevalence for women was driven by younger females. The survey also found that there has been a general increase in participation in gambling since the 2007 survey from 68 to 73%, and this increase was greater among women than men (65% in 2007 and 71% in 2010, respectively). Since gambling participation seems to be increasing faster among females than males, there may be differences in reasons for gambling requiring different treatment needs which need further examination. Furthermore services and professionals need to be prepared to treat an anticipated increase of gambling related problems in the UK.

In 2007 the British Medical Association (BMA) proposed that the NHS should provide sufficient support for gambling disorders, alongside the services it provides for drug and alcohol problems (Griffiths 2007). The theoretical review of evidence indicates that therapeutic interventions can be helpful in reducing the severity of gambling problems and improving psychological well-being in individuals with gambling related problems (Lopez Viets and Miller 1997 cited in Dowling and Cosic 2011). Current intervention options for the treatment of problem gambling in the UK include counselling, psychotherapy, cognitive-behavioural therapy (CBT), advisory services, residential care, pharmacotherapy, and combinations of these (Griffiths 1996; Griffiths and MacDonald 1999).

Yet relatively few people with gambling difficulties seek treatment (Cunningham 2005); those who develop problems in the area appear unwilling to admit these difficulties and tend to present for treatment when the severity of their difficulty drives them to treatment as a last resort (Suurvali et al. 2009), or until they have reached a crisis point (Bellringer et al. 2008). Studies from the US, Canada, Australia and New Zealand have found that between 7.1 and 29% of problem gamblers with varying degrees of difficulties seek formal help or access treatment for their problems (Cunningham 2005; Slutske 2006; Volberg et al. 2006; The Productivity Commission 2010; Ministry of Health 2007; Suurvali et al. 2008 cited in Suurvali et al. 2009). This can result in serious negative consequences for the gambler or their family, including suicidal thoughts or relationship breakdown (Carroll et al. 2011).

In addition to low numbers of individuals seeking help for gambling related problems, there are some indications that female problem gamblers are underrepresented (compared to males) in UK services. The National Problem Gambling Clinic (NPGC), which opened in 2008 and is funded partly by the Responsible Gambling Trust, is currently the only NHS multidisciplinary treatment centre for the treatment of problem gamblers with only one branch located in London. The NPGC offers a programme of individual or group cognitive-behavioural therapy (CBT). All treatment is facilitated by psychiatrists, psychologists and trainee psychologists. The programme supports the role that conditioning principles play in the maintenance of problem gambling and emphasises the way that individuals’ thinking affects their behaviour. Throughout the course of the eight sessions, individuals are given strategies to stabilise excessive gambling and once gambling is reduced, they are offered tools and techniques to help cope with urges to gamble and to minimise harm in the event of a lapse. The programme aims to teach life skills, such as mindfulness, and encourages alternative healthy activities and hobbies. Remote CBT, which is a condensed version of the treatment administered by a therapist via the telephone, is offered to individuals unable to attend treatment in person.

Between 2012 and 2013, the NPGC, received 635 referrals for treatment, with 7% of these referrals (N = 47) being from female problem gamblers (NPGC 2013). Despite the rise in numbers of female problem gamblers in the general population, the figure for female referrals has remained static since the clinic opened in 2007, prompting further investigation. Figures reveal that between March 2012 and April 2013 females were less likely to attend an offered assessment at the NPGC than men (70% compared with 81%) and more likely to miss an assessment (13% compared to 7%). Those females who attended the assessment and were then referred for treatment were much less likely to have a ‘planned’ discharge than men (27% compared to 50%) and more likely to drop out of treatment early (25% compared to 12%) (NPGC 2013). These numbers support other findings of a ‘relative absence’ of females in treatment settings where research samples of problem gamblers have previously been recruited. (Crisp et al. 2000). This is in contrast to the trend seen elsewhere in mental health where the prevalence of mild to moderate mental health conditions (mainly anxiety and depression) is thought to be similar between men and women, but women are more likely to seek help than men. In 2007 the NHS published statistics reporting that 29% of adult females were being treated for mental health disorders compared to 17% of men seeking treatment, despite similar levels (Halliwell et al. 2007).

Research has identified barriers to seeking treatment for problem gamblers, although this is not UK based and therefore should not be assumed generalisable to the UK population of problem gamblers, where presentations may be different. Furthermore, it is not gender specific, and therefore does not provide insights into barriers for female problem gamblers specifically. A literature review reporting on findings of 19 studies in five countries (Canada, Australia, New Zealand, US and Brazil) found the most commonly reported barriers were: a wish to handle the problem by oneself; shame/embarrassment/stigma; unwillingness to admit the problem; and issues with treatment itself (Suurvali et al. 2009). Other frequently reported barriers include lack of knowledge about treatment options and practical issues around attending treatment. In their quantitative study which looked at a sample of 1259 indigenous Australian adults, McMillen et al. (2004) proposed that gambling help-seeking may be reduced due to lack of awareness of gambling harms or poor access to information and gambling services. Gainsbury et al. (2014) argue that public education should aim to de-mystify the treatment process and educate gamblers about symptoms of problem gambling to reduce shame, stigma and denial and encourage help-seeking.

While there is limited research which identifies barriers for women specifically, Karter (2013) argues that women coming forward to ask for help have twice the amount of difficulties in terms of emotional, psychological and social blocks in doing so. Grant and Kim (2002) stress the importance of more aggressive and early interventions for female problem gamblers based on the ‘telescoping effect’, with quick progression of gambling problems for women. Meanwhile Wenzel and Dahl (2009) propose that treatment for female problem gamblers should focus on emotional needs, disputing the evidence base for the benefit of brief interventions. Dowling et al. (2006) proposed that CBT is an effective treatment model for female problem gamblers, based on their Australian-based, quantitative study with 19 women with Electronic Gaming Machine (EGM) problems. Yet at present the National Institute for Clinical Health and Excellence (NICE) does not offer any guidelines for the treatment of individuals with a gambling disorder. In addition to this lack of ‘best practice guidelines’, there are varying approaches for treatment for women specifically. This murky path for treatment is arguably further fueled by existing disagreements about what counts as a gambling ‘problem’ (Blaszczynski and Nower 2002).

To our knowledge there has been no published research that has phenomenologically explored the experience of treatment, or barriers to treatment for female problem gamblers in the UK. Thus, the current study, obtained via interviews, aimed to explore the lived experience of female problem gamblers who have received treatment for a gambling problem. Additionally, it was aimed at gaining access to the meanings participants attributed to the barriers of seeking and receiving treatment.

## Methods

### Participants

Participants selected fulfilled criteria for being female, speaking fluent English, being over 18 years of age and having received treatment at the National Problem Gambling Clinic (NPGC). The clinic was deemed an ideal base to conduct this study providing access to a hard-to-reach population. Recruitment of participants involved a standardised flyer given to patients at the time of their assessment. Nine female subjects who had either completed treatment or were nearing completion of treatment were invited to participate in the study.

Following screening calls, eight of the nine female volunteers agreed and were accepted as participants and completed interviews. Seven of them had received individual face-to-face CBT and one had received remote CBT. The number of sessions depended on individual circumstances.

Table 1 lists the demographics of the participants recruited for the purposes of the study. Pseudonyms have replaced real names to ensure anonymity.

**Table 1:** Female participant descriptive and demographic information

Pseudonym	Age (years)	Identified ethnicity	Relationship status	Type of gambling	Treatment duration	Stage of treatment (at time of interview)
Amy	41	British	Married	Fruit machines	5 sessions	One week since completion
Alice	55	British	Living with partner	Bookmakers, fruit machines	8 sessions (remote)	Six months since completion
Beth	42	British	Single	Slots, roulette, poker	9 sessions	One week since completion
Catherine	30	British	Separated	Arcades fruit machines and online	8 sessions	One month since completion
Diane	38	British	Living with partner	Online slots	8 sessions	One month since completion
Emma	35	British	Single	Online slots	8 sessions	One month since completion
Natalie	35	British	Single	Casino, roulette	8 sessions	Five days since completion
Jacqui	40	British	Single	Fruit machines	6 sessions	Two remaining sessions

### Procedure

Interpretative Phenomenological Analysis (IPA) is a descriptive and interpretative qualitative research method (Smith 1996, 2004). IPA was selected due to its potential to provide an ‘insider’s perspective’ through exploration, understanding and communication of the experiences and viewpoints offered by participants (Larkin et al. 2006). This correlates with the study’s aim to understand the particular phenomenon being investigated by eliciting the experiences of the ‘experts’ (Reid et al. 2005). Crucially, IPA recognises the role of the researcher in making sense of the experience of the researched (Osborn and Smith 1998; Smith 2004) acknowledging that the production of an interpretative account of a participant’s experience is co-constructed between researcher and participant (Larkin et al. 2006). The practice of reflexivity was therefore carried out throughout the research process in order to enhance awareness of the researcher’s contribution to the development of meaning-making (Nightingale and Cromby 1999). As IPA uses small sample sizes and views findings as context-dependent, statistical generalisability is not intended. However, findings can be combined with existing literature to inform practice. To ensure validity and reliability, Yardley’s (2000) four principles were consulted and applied throughout the research process. These include: sensitivity to context; commitment and rigour; transparency and coherence; impact and importance, all of which are proposed as a guideline in conducting high quality IPA.

### Interviews

Smith and Osborn’s guidelines for conducting semi-structured interviews for IPA were consulted prior to constructing the interview schedule. Designed to be collaborative in nature, emphasising the participants as primary experts and aiming to make them feel as comfortable as possible, the interview schedule started with a question that encouraged participants to recount a descriptive experience. The questions were open-ended and served as a guide for possible areas to cover, rather than leading participants towards certain responses or imposing any views on them. This style of questioning should allow truth value to emerge—which is subject orientated, not defined a priori by the researcher (Lincoln and Guba 1985). Space was left for participants to express their feelings openly and in detail. Questions were asked about participants’ experiences of treatment, barriers to treatment, positive and negative aspects of treatment, meanings they attributed to being in treatment as well as their perceptions of themselves and their gambling behaviour after treatment.

### Analysis

Analysis is an iterative and inductive cycle (Smith 2007) which involves flexibility and is expanded up until the stage of writing up; themes are constantly reworked and reorganised throughout this process. Constructing themes involved several stages. Interview transcripts were analysed using guidance on carrying out IPA (Willig and Stainton-Rogers 2008). On a case-by-case basis, each transcript was read several times. Summaries and descriptive labels were made along the margin using the participants’ words, before themes were assigned. Relationships between themes were analysed and clusters of themes formed. A cyclical activity of checking back took place to ensure that each theme was grounded in the original data. A summary table of themes, including cluster labels and illustrative quotes, was used to give structure to the data. Once relationships between themes had been established and collapsed into clusters, summary tables were created for each participant. A narrative summary based on overarching themes helped to sift out the most salient findings from the research. From here, illustrative examples of shared experiences were identified from the participants’ quotes along with those that were distinctive or contradictory. Importantly, the entire process is supported by a full ‘paper trail’, so that each step of the analytic process may be traced in detail (Yardley 2000).

## Results

The analysis of the interview transcripts generated data providing a rich and insightful portrayal of the experience of treatment for female problem gamblers. The subtheme ‘Barriers to Treatment’ is examined and expanded upon here since it was considered to warrant closer attention; perceived barriers to seeking treatment for female problem gamblers have not been explored in the UK literature and may help explain difficulties in reaching and engaging women. The findings are supported by participant quotations taken directly from the transcripts. To ensure anonymity and confidentiality, participants have been given pseudonyms and all identifying information has been either removed or altered.

Many participants referred to aspects of treatment as being ‘inaccessible’ as they described their experiences of ‘waiting’, the geographical ‘distance’, cost of travel and the sense of searching for information or help. This sense of distance, whether in space or time, seemed to encompass not only physical proximity but also an emotional distance, as all participants communicated a desire for more flexibility, convenience, and control over their treatment options. For some, the experience of waiting seemed anxiety provoking, conjuring up feelings of uncertainty and even helplessness. For others, the investment of time necessary for treatment seemed problematic, imposing and constraining. Many participants described their search for information which was not always readily available, and the disappointment that ensued. Participants appear to feel disempowered by inaccessibility issues.

Internal barriers were also identified. For many participants, denial and fear appeared to have delayed their decision to seek help. For three participants, being a woman was in itself a barrier to treatment. For others, the stigma associated with gambling as an ‘addiction’ was distressing, while for others this biological perspective provided comfort. All eight participants identified with a sense of feeling like an outsider, or as being abnormal in some way as though their gambling problems could not be understood by friends, family or treatment providers and professionals, which increased anxiety around help seeking. All participants also communicated varying degrees of ambivalence about giving up gambling, presenting some interesting challenges for treatment.

### External Barriers to Treatment

For Diane there was a strong theme of waiting and time, which ran throughout her entire narrative, from her unsettling experience of waiting for treatment, to a sense of losing time, boredom and an element of surprise or suddenness relating to her gambling, to her perception of time as a healer with regard to loss and pain she had experienced. She explains:

I’d asked for treatment earlier but…because resources are limited you can’t go straight away, so it was quite a few probably months had elapsed before actually getting it… When you’re just waiting it is one of those things where you just need it now, like that’s usually the point where you ask for help. By the time I got to the sessions I was already urm I was in a period of abstinence. (Diane)

For Diane, waiting seems to evoke anxiety, frustration and even desperation, with an element of uncertainty, ‘you can’t know’, and a sense of being disempowered, ‘you can’t go straight away’, and ‘you just need it now’, all of which is narrated in the second person as she distances herself from the experience. She recalls ‘just waiting’ illustrating her preoccupation and sense of powerlessness. She explains she was already abstinent ‘by the time’ she was offered help, communicating that she was let down and treatment came too late, highlighting the importance of timing for intervention. This might also lead the reader to wonder how she achieved this abstinence. She also describes the significance of her free time:

It’s going to take your time and so on…like I’m going to have to….I know it sounds silly but I’m going to have to fill out some forms and then make my way to X or give X hours a week.

For Diane the investment of time for treatment is an additional barrier to accessing help, as she describes ‘it’s going to take your time’. Filling out forms and travelling to the clinic also appear. To be burdensome or imposed, as she reflects ‘I’m going to have to’. Like Diane, this sense of waiting for therapy and then making the commitment in time proved problematic for Emma, who comments:

There was a long waiting list and then unfortunately three months on the trot I lost everything each time so the only negative thing is that this isn’t, there’s only one clinic with a waiting list. (Emma)

Both Emma and Diana articulated their understanding about practicalities within the service with regard to the waiting list, yet their disappointment is clear, as Emma acknowledges ‘unfortunately three months on the trot I lost everything’.

For Catherine and Alice, the journey from the clinic was an obstacle standing between themselves and their efforts to seek treatment, creating a sense of distance for them:

There was an after-group but unfortunately it was in the evenings and as it was in London I don’t live that near and I wasn’t able to attend….so that was the downside, I feel that I needed further help but I couldn’t access it because of how far away it was and because it was late. I think something in the day would have been a lot easier. (Catherine)

For Catherine, who was the only mother among the eight participants, distance and timing of sessions served as a barrier to accessing the optional monthly post-treatment group. She needs more flexibility and convenience, with ‘day’ sessions that might accommodate her schedule. Childcare posed a further difficulty, as she explains:

I think being a single female has because of the childcare side of it and also I’ve got a very big fear of separation anxiety over my children so….unless there was something available at meetings I wouldn’t want to leave them with anybody. (Catherine)

Catherine views herself as being unsupported as a ‘single female’, highlighting a sense of unavailability of help, yet in the same sentence she acknowledges her ‘separation anxiety’, which in itself acts as a barrier as Catherine becomes torn between getting help for herself and the anxiety she feels around leaving her children with others. An interesting juxtaposition emerges since her children have been both a motivating factor and an obstacle for Catherine to receive help, while she is unable to tolerate the isolation and overwhelming loneliness of her inner world.

For Alice, who works in a supermarket on minimum wage, the distance, cost and wait were also a barrier for her to access support; it is as though she feels unwelcome by the prospect of travelling so far, but she was offered remote therapy, on the telephone, which met her needs. She illustrates this here:

I live here, which is far away from there and it’s expensive to travel to London if you drive in a car that costs money as well to park. (Alice)

Some of the females in the study reflected on their discovery of the clinic, communicating that there was limited information available and other professionals were not aware of the service. Natalie explains:

To be honest, I didn’t realise there was an NHS thing … I don’t think many people know about it… I went to the doctor a few times like I said about the suicide or gambling; they never advised me about it…to be honest they just prescribed me antibiotics. (Natalie)

These narratives highlight several accessibility issues in treatment, highlighting the significance of time, waiting, distance, childcare and available information and support.

### Internal Barriers to Treatment

A number of ‘internal barriers’ also emerged from the participants’ narratives, and they appear to have prevented females from accessing support sooner. These range from denial of the problem, fear, stigma and ambivalence. Several participants admit to non-acknowledgement of their problem, or attempting to control it or manage it themselves prior to feeling desperation or hitting ‘rock bottom’ and seeking help. This is illustrated here by Emma, who explains:

I didn’t think I had a big problem, but clearly I did, that was me in denial and me concentrating on other people’s issues, I felt like I was I don’t know just taking the limelight off me and was trying to help others. (Emma)

Emma felt she was able to focus on others’, rather than her own needs, and in this way continued to avoid reality. This provides a glimpse into Emma’s inner world, where she hides from the ‘limelight’ and takes refuge from feeling needed or being able to help or please others. For other women, opening up to others and in this way placing their trust in them, and connecting with difficult feelings such as shame, guilt, embarrassment and fear acts as another barrier to treatment. Diane explains:

I’m less open, I internalise a lot…it’s just a personality style you know I keep stuff in a lot and some people are much more, urm, not open but just talk about themselves more and I just don’t really do that in daily life so that was a challenge…I don’t go to the doctor unless I’m ill, unless it’s really bad, because it’s quite a rigmarole to go, so there is a barrier, the other is sort of a responsibility that I should sort it out myself. (Diane)

Here Diane reflects on what held her back from seeking help sooner; she acknowledges only asking for help when things are ‘really bad’, she acknowledges the ‘sense of responsibility’ she feels to solve problems herself, and then there is perhaps also a sense of belief in her ability to do so. Natalie also communicates how difficult her initial session was, as she illustrates her concerns about what goes on in therapy and its quality or effectiveness:

I kind of had the opinion that it wouldn’t work and how can it work and I was also a little bit scared that it would actually change my opinions on things though it sounds stupid…(laughs) you know it was behavioural therapy I was thinking I don’t want it to actually change my (laughs) brain but I’d like it to stop me gambling but apart from that I don’t want anything like my personality to change. (Natalie)

Natalie articulates the uncertainty, doubt, mistrust, scepticism and fear she felt about therapy prior to attending, as she describes her concerns that ‘it wouldn’t work’, ‘how can it work?’ accompanied by her fear of losing control at the prospect of placing her inner world in the hands of a stranger and relinquishing some control, in case this might ‘change her opinions’, ‘brain’ or even ‘personality’. For Natalie, who admits she does not like feeling out of control, it was a big step to put her faith in professionals. As she disclosed her concerns, she appeared embarrassed, frequently laughing, letting me know ‘it sounds stupid’ as she fiddled with her hair and averted her eyes. It seemed difficult for her to communicate her fear, yet she was able to work through this and her relief was evident.

The data also reveal an interesting mixture of experiences regarding sociocultural norms and expectations. Most of the females acknowledged some type of norm or expectation, but some felt more influenced by these than others, and only four had experienced these in relation to treatment, describing how being a woman had impacted on their ability to access treatment, with limited options available for women. Amy sees the norm for gambling as being male-dominated and acknowledges the impact of this in terms of her sense of self and how she feels others perceive her:

You can see all the men in the bookies, down the horse races, you very rarely see females playing fruit machines with the men in the bar. I think it’s predominantly a male thing, which is why as a female doing it you feel even worse, you think there’s something wrong with me. (Amy)

For Amy, the fact that she does not conform to the UK norm appears to be a great source of shame as she feels abnormal, as though there is something ‘wrong’ with her. This may be a product of societal influence and social construction but as she discloses her reluctance to allow her parents into her world of gambling, ‘I wouldn’t want my parents to know’, it appears these values may be deeper rooted on a cultural or familial level. As Amy has communicated her shame and embarrassment about her minority status, she imagines feeling ‘out of her depth’ at the idea of sitting in a treatment group with male gamblers, as though she does not deserve to be there, as she describes:

I think men would dominate it and I would feel a bit shy you know because they are more common and we are a bit rarer. I would feel a bit out of my depth, out of control, out of my depth, different. (Amy)

While Amy appears to feel under considerable pressure to conform to ideals and social norms, feeling intimidated by the idea of a mixed support group, Beth and Catherine communicate their struggle to access adequate support because of their gender. For these women, the rejection they have experienced while trying to access services appears more problematic. Beth elaborates:

I’ve never seen a female in recovery in a GA meeting…initially they look at you and they said you’re female you should be in the partner’s room, and I’m not a partner, I’m a gambler, I need to be in this room, so you get rejected a lot. (Beth)

Throughout Beth’s narrative she portrays her long, uphill search to find help. She experiences being cast aside and dismissed at GA, and it seems that this rejection feels familiar for her as she protests that she wants to stay in the room and describes the way ‘you get rejected a lot’, distancing herself from the painful experience by narrating in the second person. Not only does she experience rejection from society but also from treatment groups where she is turned away. Like Beth, Catherine feels she had some difficulty accessing treatment due to her gender, but for her it was the childcare access that proved to be the main barrier to treatment as previously discussed. However she also makes a more general observation about treatment availability for gambling, comparing it to drug and alcohol addictions.

You can get help for drugs and alcohol but gambling there isn’t much help in London. (Catherine)

These experiences appear to be in contrast to those of younger participants, or participants who have been gambling for less time, and they could be interpreted as a contextual difference relating to age and changing cultural norms or treatment options. Alice does not perceive her gender to have impacted on her treatment experience, despite being prompted. Alice explains:

No, why should it (ha) all sorts of people gamble, don’t they? There’s no stereotype, is there? I mean look at them - they’re all lonely people, I think? If you look at gamblers they are quite lonely? (Alice)

In this short extract, Alice emphasises that ‘all sorts of people gamble’, denying that there is any stereotype for gambling in relation to gender, yet she appears to relate more to the ‘loneliness’ that she believes is shared by gamblers. Emma acknowledges a ‘stigma’ surrounding gambling but she does not seem to feel ashamed to be gambling as a result of her gender. Like Catherine, she compares gambling addiction to drug and alcohol addiction. For her, the frustration appears to be around feeling misunderstood. She explains:

He (boyfriend) wasn’t supportive in any way even though he’s an alcoholic, even though he’s got issues and I’ve supported him he couldn’t understand, it feels like there’s a stigma on gambling even in comparison to, I got told you’re worse than a crack addict. (Emma)

Emma’s sense of not feeling understood is echoed and repeated by all the participants in this study and dominated as a theme. Participants described feeling as though they were unique, different, special or abnormal in some way, viewing themselves as an outsider in a range of contexts. These descriptions communicated an expectation that participants would fail to be understood, seen or heard by others, portraying an isolated existence in which they are rejected, left to cope alone, without validation. They appear to feel alone with an uncommon problem, which for some acts as an obstacle to accessing help.

I think the hardest thing to cope with is that it’s not understood very well. One social worker said to my face that she doesn’t see why I do it either, which hurt a lot. (Catherine)

For Catherine, the ‘hardest part’ of being a problem gambler is not being understood, even by professionals, and this demonstrates how difficult this is for her. She uses the word ‘hurt’, revealing how others’ lack of understanding about addiction has felt for her. This fear of being misunderstood by others is echoed by Emma, who emphasises her concern about being placed into a box marked ‘gambler, rather than being treated as a person. She illustrates this here:

Even though we have all got the same thing, we’re all compulsive gamblers…we might have different gambling issues with different reasons and I think something that might work for the lady sitting next to me might not, you know, it might not work for you know, yeah it might not work for me what works for the next person (Emma)

The importance of recognising individual difference in herself and in others is clear throughout Emma’s narrative. As she explains her comment ‘even though we have all got the same thing’ she emphasises that she is more than just a ‘diagnosis’. This is highlighted by other participants who communicate the need for a more personalised treatment experience. For example, several participants express the desire to have a therapist with a shared experience. Jacqui explains:

I’m sure if you were going to go to a bereavement counsellor they would have experience of bereavement, so why should you be a gambling counsellor and not have experience of gambling…I feel like they’re talking kind of like from a text book, I mean they’re very good at what they do I’m not saying they’re bad but they haven’t got that little tiny thing that only a gambler would know….yeah I really don’t think you can fully understand something until you’ve had it. (Jacqui)

Jacqui seems frustrated that her therapist did not have experience of gambling, as she poses the question ‘why should you be a gambling counsellor and not have experience?’, articulating that her therapist was not able to make a real connection with her since it felt as if she were being spoken to from a ‘text book’, as though her therapist were intent on box ticking rather than getting to know Jacqui. She articulates her sense of not being understood.

Another internal barrier to treatment that was identified among participants was ambivalence and inner conflict. This is encapsulated by Amy, who still fantasises about gambling and seems resigned to the idea that it will always remain a part of her. She reflects on this here:

I still dream about it, urm, about the gambling…I don’t think I’ll ever get over it. (Amy)

Here Amy points out the discrepancy she experiences between the reality of treatment and what is happening in her inner world. This sense of ambivalence is a common theme among all participants, many of whom communicated that gambling is a part of who they are, and the sense of loss they experience without it. Jacqui sums this up here:

I am very shy, but then I was never shy in gambling…I do miss the confidence I had with it, I could walk into any casino and feel confident….I don’t think I’ll ever stop completely’. (Jacqui)

For Jacqui, letting go of gambling also signifies a loss of ‘confidence’. She felt accepted as a gambler; protected and validated. It is as though the casino provided a temporary respite with a new sense of ‘confidence’.

The narratives in this section demonstrate internal and external barriers to treatment, highlighting the complexity of female problem gamblers’ journey to treatment which appears to be fraught with practical accessibility issues and inner conflict.

## Discussion

The present research provides novel insights from treatment-seeking female problem gamblers in the UK, highlighting some of the specific difficulties they experience throughout their treatment journey. Results suggest that women experience time, waiting, distance, cost and lack of information as practical barriers to treatment. Health care professionals may also be a barrier to treatment, preventing women from accessing services. The authors argue that unless professionals such as general practitioners are educated around the signs of problematic gambling, what to look out for and where to signpost, women may be unaware of having a gambling problem and may be failed in this way.

Internal barriers which were prevalent and meaningful in the women’s narratives included denial of the problem, fear, ambivalence, stigma, shame, and feeling misunderstood. This supports findings from previous, non-gender specific studies from the US and Australia (Suurvali et al. 2009; Gainsbury et al. 2014), suggesting these barriers may be exist for both men and women. However, more specific information is not available to clarify which barriers were more specific to the men or women who participated in these studies; therefore it is difficult to make any links.

Overall findings from this study suggest that service providers should consider alternative means of engaging female problem gamblers in treatment, which take into account clients’ resources, including their psychological mindedness, readiness to engage and openness to trying new things. Limitations associated with the NHS must be also taken into consideration. For example, waiting lists cannot easily be reduced when there is an increasing demand for services and lack of funding. However, this raises the question of whether more services are required to better meet the needs of problem gamblers. Further light can be shed on this by Rigbye and Griffiths (2011), who found that 97% of the NHS Trusts did not provide any service to treat people with gambling problems, arguing that treatment centres are not geographically accessible, and in this way the treatment needs of problem gamblers are not being met. This highlights the need for more research in this area and more collaboration between professionals working with problem gamblers in order to gain a better understanding of current difficulties.

Results indicate that online therapy, helplines and forums may be beneficial for women to overcome some of the practical accessibility issues, such as time, distance, cost and childcare issues, supporting findings from recent studies that online support services appeared to be more favoured by women than any other service (Wood and Griffiths 2007; Rodda and Lubman 2013). These types of services aim to address barriers to treatment such as shame and stigma, geographic isolation and hours of operation (Rodda and Lubman 2013), and may therefore provide alternative means of engaging hidden populations who need help.

A further recommendation based on these findings is that treatment providers should be mindful of the enormous challenges women have faced and overcome. These include stigma, fear, rejection and their sense of feeling misunderstood, or abnormal. In order to address this we propose providing a restorative relational experience in the therapeutic encounter, remaining person-centred rather than disorder-centred and providing a bespoke, individualised treatment experience that takes context into account.

It also seems important to establish what conditions need to be present for an effective intervention, and how to help women overcome internal struggles to enable them to access and utilise available treatment. Motivational interviewing (MI) (Miller and Rollnick 2002) is a ‘person centred’ way of addressing ambivalence about change, in which the therapist may facilitate the client’s expression of both sides of their ambivalence and guide them towards a more acceptable resolution which may assist them in triggering change; in this way it is a collaborative approach to help strengthen a person’s own motivation and commitment to change. Hodgins et al. (2001) showed participants who received a motivational telephone intervention plus a self-help workbook were more likely to be classified as improved for gambling problems than those who received only the workbook with no intervention. This supports the idea of a motivational intervention, yet treatments for gambling problems are not well established and there is limited research that demonstrates which types of treatments are effective. More guidelines and research on treatments may serve to decrease uncertainty among practitioners or gamblers themselves as to treatment outcomes. This may involve consultation with NICE in order to establish more universal guidelines for treatment.

Results suggest that more efforts are needed to further raise awareness of problem gambling and options for treatment of problem gambling, such as media-based campaigns and collaboration and discussions with GPs, social services and other professionals who are likely to come into contact with problem gamblers. This is so as to increase awareness of the problem and treatment options. We would also like to propose increased collaboration between dedicated treatment centres for problem gamblers to ascertain whether similar patterns are being observed to increase learnings. This is in line with Gainsbury et al.’s suggestion that public education is needed to ‘demystify’ the treatment process (Gainsbury et al. 2014).

### Limitations and Further Directions

It is important to acknowledge several limitations. This was a small study and was reliant on volunteers, self-report and retrospective assessment of barriers to help-seeking. Only focusing on one NHS outpatient clinic with a specific way of working may not have yielded analogous results to those of other treatment centres. Since no other study of this kind currently exists in the UK it is not possible to ascertain whether findings are typical, therefore it is prone to issues of generalisability. More research is needed in the same area in order to substantiate the current study and enhance reliability. It would be useful to conduct the study on a larger scale, across a range of problem gambling treatment centres in the UK as well as non-specialist settings to determine whether perceptions of barriers to treatment differ according to other elements. This study could also be done on an international scale, taking into account contextual factors for females. This would add to the findings from this small-scale study and inform treatment providers.

The absence of a male comparison group precludes forming conclusions on gender differences in the experience of treatment for female problem gamblers. While our intent was to focus on females, to address a gap in the literature, in doing so, we have missed out on additional information which could have been provided by males. Future research on males’ perspectives would be valuable either in conjunction with, in comparison to, or separately from studies investigating female treatment experiences and barriers.

Participants who volunteered were in treatment and felt able to speak about their difficulties, and therefore they had recognised and admitted gambling posed a problem for them. Since the method relied on self-report, it is vulnerable to weaknesses, including faulty memory, factual errors and self-presentation biases.

Furthermore, those gamblers who had not yet taken these steps or struggled to articulate their difficulties were not represented in the data. Therefore gaps remain in research on problem gambling among those who may be more socially, physically or linguistically isolated. In addition it would be useful to speak with problem gamblers who had dropped out of treatment.

Several methodological limitations were also identified. While IPA can provide a detailed picture of a participant’s experience, reflecting the complexity of this experience and its embedded nature in the individual lifeworld, it cannot explain social processes. A grounded theory study may provide a greater understanding of the high attrition rates at the NPGC, explaining some of the processes behind this phenomenon, and it could go further to explain what it is that stops females from coming forward for help, generating theory. Unlike IPA, grounded theory claims to be generalisable based on theoretical saturation.

A strength of this study was the richness of the data illicited from participants. Since questions were open ended, responses can provide a vivid picture of respondents’ experiences, rather than forcing choice or categories via quantitative methods such as questionnaires. Open-ended interviews can help identify the particular aspects of barriers which are most troubling to this subgroup of problem gamblers, with the emphasis placed upon particular barriers and how they inhibit help-seeking.

Future research could explore barriers to treatment on a deeper level, for example investigating whether obstacles acquire special meaning in certain situations or subgoups of women including age, marital status, employment, religion, class and area. It could also examine specific barriers more rigorously. Future research could also investigate attrition in female problem gamblers on both a national and international scale, however for a detailed exploration of this topic, interviews or questionnaires would need to be conducted with women who had dropped out of treatment or those who had not yet engaged in treatment. Gaining access to this hidden population is likely to be more complicated. The recruitment sample pool and methods would need to be expanded to online forums. Additional research could examine awareness of professional sources of help and help-seeking behaviours among gamblers and problem gamblers in the UK. Finally, future research should also focus on other hard to reach populations which show increasing participation in gambling, including adolescent problem gamblers.

## Conclusion

The field of problem gambling is a complex area where further research is needed to establish whether there is a more effective way to reach and engage women with problems in this area. Despite the relative variance of treatment episodes, gambling preferences, age and demographics of the sample, there was considerable consensus regarding barriers to treatment. Fundamentally for this cohort of female problem gamblers, overlapping internal and external barriers appeared to have delayed their decision to seek help sooner. These barriers to help-seeking can be addressed with changes to the way help is offered, perceived and delivered. While funding and resources within the NHS must be taken into account, it is argued that the therapeutic encounter itself may be instrumental in breaking down some of the barriers to support and engage women. Attention should be paid to providing individualised, bespoke treatment experiences which provide a ‘secure base’ founded on a strong therapeutic alliance. More research is needed to expand our understanding of which treatment models are most effective. In addition, public education with regard to problem gambling should be stepped up to increase awareness, reduce stigma and encourage help-seeking among those with problems in this area.

## References

[CR1] Bellringer, M., Pulford, J., Abbott, M., DeSouza, R., & Clarke, D. (2008). Problem gambling-barriers to help-seeking behaviours. Auckland. Retrieved from: http://aut.researchgateway.ac.nz/bitstream/handle/10292/2014/Report%20final%2010%20September%202008.pdf?seq.

[CR2] Blaszczynski A, Nower L (2002). A pathways model of problem and pathological gambling. Addiction.

[CR3] Carroll A, Davidson T, Marsh D, Rodgers B (2011). Help-seeking and uptake of services amongst people with gambling problems in the ACT.

[CR5] Crisp BR, Thomas SA, Jackson AC, Thomason N, Smith S, Borrell J, Ho W, Holt TA (2000). Sex differences in the treatment needs and outcomes of problem gamblers. Research on Social Work Practice.

[CR6] Cunningham JA (2005). Little use of treatment among problem gamblers. Psychiatric Services.

[CR7] Dowling N, Cosic S (2011). Client engagement characteristics associated with problem gambling treatment outcomes. International Journal of Mental Health and Addiction.

[CR8] Dowling N, Smith D, Thomas T (2006). Treatment of female pathological gambling: Efficacy of a cognitive-behavioural approach. Journal of Gambling Studies.

[CR10] Gainsbury S, Hing N, Suhonen N (2014). Professional help-seeking for gambling problems: Awareness, barriers and motivators for treatment. Journal of Gambling Studies.

[CR11] Grant JE, Kim SW (2002). Gender differences in pathological gamblers seeking medication treatment. Comprehensive Psychiatry.

[CR12] Griffiths MD (1996). Pathological gambling: A review of the literature. Journal of Psychiatric and Mental Health Nursing.

[CR13] Griffiths MD (2007). Gambling addiction and its treatment within the NHS: A guide for healthcare professionals.

[CR14] Griffiths MD, MacDonald HF (1999). Counselling in the treatment of pathological gambling: An overview. British Journal of Guidance and Counselling.

[CR15] Halliwell, E., Main., L, & Richardson, C. (2007). *The fundamental facts, latest facts and figures on mental health*. New Delhi: Mental Health Foundation. Retrieved from http://www.mentalhealth.org.uk/content/assets/PDF/publications/fundamental_facts_2007.pdf?view=Standard.

[CR16] Hodgins DC, Currie SR, El-Guebaly N (2001). Motivational enhancement and self-help treatments for problem gambling. Journal of Consulting and Clinical Psychology.

[CR17] Karter L (2013). Women and problem gambling: Therapeutic insights into understanding addiction and treatment.

[CR19] Larkin M, Watts S, Clifton E (2006). Giving voice and making sense in interpretative phenomenological analysis. Qualitative Research in Psychology..

[CR20] Lincoln YS, Guba EA (1985). Naturalistic inquiry.

[CR21] McMillen J, Marshall D, Murphy L, Lorenzen S, Waugh B (2004). Help-seeking by problem gamblers, friends and families: A focus on gender and cultural groups.

[CR22] Miller WR, Rollnick S (2002). Motivational interviewing: Preparing people for change.

[CR23] Ministry of Health (2007). Preventing and minimising gambling harm.

[CR24] National Problem Gambling Clinic (NPGC). (2013). Data retrieved from NPGC database.

[CR26] Nightingale D, Cromby J (1999). Social constructionist psychology.

[CR27] Osborn M, Smith JA (1998). The personal experience of chronic benign lower back pain: An interpretative phenomenological analysis. British Journal of Health Psychology.

[CR29] Potenza MN, Steinberg MA, McLaughlin SD, Wu R, Rounsaville BJ, O’Malley SS (2001). Gender-related differences in the characteristics of problem gamblers using a gambling helpline. American Journal of Psychiatry.

[CR32] Reid K, Flowers P, Larkin M (2005). Exploring lived experience. The Psychologist.

[CR33] Rigbye J, Griffiths M (2011). Problem gambling treatment within the British National Health System. International Journal of Mental Health Addiction.

[CR35] Rodda S, Lubman D (2013). Characteristics of gamblers using a national online counselling service for problem gambling. Journal of Gambling Studies.

[CR36] Slutske WS (2006). Natural recovery and treatment-seeking in pathological gambling: Results of two US national surveys. American Journal of Psychiatry.

[CR37] Smith JA (1996). Beyond the divide between cognition and discourse, using interpretive phenomenological analysis in health psychology. Psychology and Health.

[CR38] Smith JA (2004). Reflecting on the development of interpretative phenomenological analysis and its contribution to qualitative psychology. Qualitative Research Psychology.

[CR39] Smith JA (2007). Hermeneutics, human sciences and health: Linking theory and practice. International Journal of Qualitative Studies on Health and Well-Being.

[CR41] Suurvali H, Cordingley J, Hodgins D, Cunningham J (2009). Barriers to seeking help for gambling problems: A review of the empirical literature. Journal of Gambling Studies.

[CR43] The Productivity Commission. (2010). Retrieved from http://www.pc.gov.au/inquiries/completed/gambling-2009/report.

[CR44] Volberg, R. A., Nysse-Carris, K. L., & Gerstein, D. R. (2006). *2006 California problem gambling prevalence survey*. Final report. Chicago: National Opinion Research Centre (NORC) at the University of Chicago.

[CR45] Wardle, H., Moody, A., Spence, S., Orford, J., Volberg, R., Jotangia, D., et al. (2011). *British gambling prevalence survey*. London: National Centre for Social Research. Retrieved from http://www.gamblingcommission.gov.uk/PDF/British%20Gambling%20Prevalence%20Survey%202010.pdf.

[CR46] Wardle, H., & Seabury, C. (2013). ‘Gambling behaviour’ in Craig, R., Mindell, J. (Eds.), *Health survey for England 2012*. Leeds: Information Centre for Health and Social Care. Retrieved from http://www.hscic.gov.uk/catalogue/PUB13218/HSE2012-Ch7-Gambling-behaviour.pdf.

[CR47] Wenzel H, Dahl A (2009). Female pathological gamblers. A critical review of the clinical findings. International Journal of Mental Health and Addiction.

[CR49] Willig C (2013). Introducing qualitative research in psychology.

[CR50] Willig C, Stainton-Rogers W (2008). The SAGE handbook of qualitative research in psychology.

[CR51] Wood R, Griffiths MD (2007). A qualitative investigation of problem gambling as an escape-based coping strategy. Psychology and Psychotherapy: Theory, Research & Practice.

[CR52] Yardley L (2000). Dilemmas in qualitative research. Psychology and Health.

